# Predictable biomarkers of developing lymphoma in patients with Sjögren syndrome: a nationwide population-based cohort study

**DOI:** 10.18632/oncotarget.15100

**Published:** 2017-02-04

**Authors:** Yu-Hsiang Chiu, Chi-Hsiang Chung, Kuen-Tze Lin, Chin-Sheng Lin, Jia-Hong Chen, Hsiang-Cheng Chen, Ren-Yeong Huang, Chi-Tsung Wu, Feng-Cheng Liu, Wu-Chien Chien

**Affiliations:** ^1^ Department of Internal Medicine, Division of Rheumatology/Immunology/Allergies, Tri-Service General Hospital, National Defense Medical Center, Taipei, Taiwan; ^2^ School of Public Health, National Defense Medical Center, Taipei, Taiwan; ^3^ Department of Radiation Oncology, Tri-Service General Hospital, National Defense Medical Center, Taipei, Taiwan; ^4^ Department of Internal Medicine, Division of Cardiology, Tri-Service General Hospital, National Defense Medical Center, Taipei, Taiwan; ^5^ Department of Internal Medicine, Division of Hematology/Oncology, Tri-Service General Hospital, National Defense Medical Center, Taipei, Taiwan; ^6^ Department of Periodontology, Tri-Service General Hospital, National Defense Medical Center, Taipei, Taiwan; ^7^ Department of Oral and Maxillofacial Surgery, Tri-Service General Hospital, National Defense Medical Center, Taipei, Taiwan; ^8^ Department of Medical Research, Tri-Service General Hospital, National Defense Medical Center, Taipei, Taiwan

**Keywords:** Sjögren syndrome, non-Hodgkin's lymphoma, diffuse large B-cell lymphoma, immunosuppressive agents, lip biopsy

## Abstract

Sjögren syndrome (SS) is commonly known to be correlated with lymphoma. This study included 16,396 individuals in the SS cohort and 65,584 individuals in the non-SS cohort, all of whom were enrolled in the Taiwan National Health Insurance database between 2000 and 2010. We evaluated the risk factors of non-Hodgkin's lymphoma (NHL) in the primary SS cohort by applying a Cox multivariable proportional-hazards model. We increased the correlation of patients with SS and NHL, with an adjusted HR of 4.314 (95% CI 2.784 – 6.685). Of the 16,396 SS patients, 66 individuals had salivary gland slices without NHL development, while the other 16,330 individuals that did not have salivary gland slices revealed 30 individuals that developed NHL. Of the 16,396 SS patients, 128 individuals underwent immunomodulator agent therapy (including hydroxychloroquine, azathioprine, cyclosporine, methotrexate, and rituximab) without NHL development. None of the 30 individuals that developed NHL from SS received immunomodulator agents. We found that patients with SS were at an increased risk of developing NHL, with the most common NHL subgroup being diffused large B-cell lymphoma. SS patients who were candidates for salivary gland slices or immunomodulator agents were associated with a lower risk of developing lymphoma over time. We recommend that patients at a higher risk upon diagnosis of SS receive close follow-up and aggressive treatment.

## INTRODUCTION

Sjögren syndrome (SS) is an autoimmune disease that involves the exocrine glands and epithelia inflammation with lymphocyte infiltration, particularly in salivary and lacrimal glands, which results in inflammatory cytokines, while the activation of B lymphocytes results in autoantibody production. The most common cause of mortality in SS patients is cardiovascular disease, followed by malignancies and infections [1]. Hematologic malignancy is the most fatal complication experienced by SS patients, with an eight-fold risk of mortality when compared to normal population [2, 3]. Non-Hodgkin's lymphoma (NHL) occurs in approximately 2.7 - 9.8% of SS patients, with a greater risk than the normal population (Table 1); such patients are also at an increased risk for other autoimmune diseases [4–24]. The B-cell type was mostly seen as mucosa-associated lymphoid tissue lymphoma, nodal marginal zone lymphoma, and diffuse large B-cell lymphoma [5, 9, 21, 25].

**Table 1:** Previous study of Sjögren's syndrome related to lymphoma

No.	Year	Study design	The relation of lymphoma to Sjögren's syndrome	Reference (First Author)
1	1964	Cohort of 38 patients with Sjögren's syndrome	10.5 % (4/38) developed lymphoma	Talal, N.
2	1978	Cohort of 136 women with sicca syndrome	5.1 % (7/136) developed non-Hodgkin's lymphoma	Kassan, S. S.
3	1992	Cohort of 120 patients with primary Sjögren's syndrome	6.7 % (8/120) developed non-Hodgkin's lymphoma	Pavlidis, N. A.
4	1996	Cohort of 103 patients with primary Sjögren's syndrome from 1986 to 1991	6.8 % (7/103) developed lymphoma	Tzioufas, A. G.
5	1997	Cohort of 676 patients with primary Sjögren's syndrome, 709 with secondary Sjögren's syndrome	The standardized incidence ratio of non-Hodgkin's lymphoma was 8.7 for primary Sjögren's syndrome and 4.5 for secondary Sjögren's syndrome.	Kauppi, M.
6	1997	Sjögren's syndrome detected among 33 newly diagnosed untreated patients with non-Hodgkin's lymphoma	6 % (2/33) detected Sjögren's syndrome, both were male with lung and stomach non-Hodgkin's lymphoma	Andonopoulos, A. P.
7	1997	Cohort of 331 patients with Sjögren's syndrome	2.7 % (9/331) developed non-Hodgkin's lymphoma	Valesini, G.
8	1999	Cohort of 100 patients with primary Sjögren's syndrome	3 % (3/100) developed non-Hodgkin's lymphoma, 2 were parotid, and 1 was retroperitoneal	Davidson, B. K.
9	2000	Cohort of 261 patients with primary Sjögren's syndrome	3.4 % (9/261) developed lymphoma	Skopouli, F. N.
10	2001	Cohort of 111 patients with primary Sjögren's syndrome diagnosed in 1977-1992	2.7 % (3/111) developed non-Hodgkin's lymphoma, with standardized incidence ratio 13	Pertovaara, M.
11	2005	Meta-analysis 5 cohort	2.3 % (30/1323) developed lymphoma, the standardized incidence rate was 18.8	Zintzaras, E.
12	2006	Retrospective evaluation 112 patients with primary Sjögren's syndrome	9.8 % (11/112) developed lymphoma (either before or after development of pSS),	Lazarus, M. N.
13	2006	Population-based case-control study, 3055 NHL patients and 3187 matched control	Primary Sjögren syndrome had an odds ratio of 6.1 in non-Hodgkin's lymphoma, subtype mentioned in article	Smedby, K. E.
14	2008	Pooled analysis of individual data from 8 case-control studies that reported history of Sjögren's syndrome in patients with non-Hodgkin's lymphoma,	0.6 % (52/8178) of patients with non-Hodgkin's lymphoma had a history of Sjögren's syndrome, 0.3 % (23/8176) had primary Sjögren's syndrome	Ekstrom Smedby, K.
15	2009	Cohort of 536 patients with primary Sjögren's syndrome from 1981 to 2008	7.4 % (40/536) developed lymphoma, subtype mentioned in article	Baimpa, E.
16	2011	Cohort of 445 patients with primary Sjögren's syndrome from 1985 to 2009	4 % (18/445) developed lymphoma	Martel, C.
17	2011	Cohort of 244 patients with primary Sjögren's syndrome	4.5 % (11/244) developed non-Hodgkin's lymphoma, subtype mentioned in article	Solans-Laque, R.
18	2011	Cohort of 175 patients with primary Sjogren's syndrome and with a median onset of 7 years following	4% (7/175) developed non-Hodgkin's lymphoma	Theander, E.
19	2012	Cohort of 584 patients with Sjogren's syndrome from 1980 to 2010	The prevalence was 3.6% (6/163) in 1995, 4.3% (17/399) in 2000, and 7.5% (40/536) in 2008, subtype mentioned in article	Voulgarelis, M.
20	2012	Cohort of 7852 patients with primary Sjogren's syndrome from 2000 to 2008	Standardized incidence ratio of 7.08 in female patients compared with the general population	Weng, M. Y.
21	2014	Retrospective evaluation 1115 patients with primary Sjogren's syndrome	4.5 % (50/1115) developed non-Hodgkin's lymphoma	Baldini, C.

Currently, both clinical and laboratory features have been associated with increasing risks of developing NHL from SS; such features include major salivary gland enlargement, splenomegaly, lymphadenopathy, purpura, low complement level, especially C4, monoclonal gammopathy, cryoglobulinemia, neutropenia, and lymphopenia upon diagnosis of SS [2, 16, 21, 25–31]. In 2002, the American-European consensus group (AECG) revised the classification criteria of SS [32]. The six criteria groups are classified as two groups of clinical symptoms (ocular and oral symptoms) and four groups of objective findings (ocular signs, histopathology, salivary gland objective diagnostic tests, and autoantibodies). A diagnosis is made when four (must include histopathology or serology) of the six criteria or three of the four objective findings are met. Of these criteria, histopathology from lip biopsies of minor salivary glands was the most invasive procedure. Seronegative patients, those with negative results on anti-Ro/SSA or anti-La/SSB antibodies, or those with clinical symptoms and signs are candidates for lip biopsy to meet the criteria. SS is associated with B-cell dysfunction, with a higher incidence and higher serum levels of the B-cell activating factor [33, 34].

The purposes of this study are to provide an overview of the risk of NHL and its subtypes in SS patients and to recognize the related risk factors and correlations with other preexisting comorbidities, such as diabetes, hypertension, depression, stroke, dementia, or chronic kidney disease. We hypothesized that reduced B lymphocyte activity, including seronegative SS (negative on anti-Ro and anti-La), and patients that received immunomodulator agents, could reduce the subsequent risk of lymphoma. We also attempted to identify whether the risk was reduced in SS patients by studying candidates from lip biopsy who were found to be seronegative upon their diagnosis of SS.

## MATERIALS AND METHODS

### Data source

The National Health Insurance (NHI) program has been the single payer providing comprehensive medical care for all people in Taiwan since March 1, 1995. As of June 2015, 23,644,464 people were enrolled in NHI through 817,902 insurance registration organizations [35]. The National Health Insurance Research Database (NHIRD) consists of the claims records from Taiwan's universal NHI program, which was established by the National Health Research Institute (NHRI). All personal identification information is encrypted prior to the data being released for research purposes in order to protect patient privacy. The NHIRD has been described in detail in previous studies [36, 37].

In this study, we used a NHIRD subset that contained healthcare data, including files from the Longitudinal Health Insurance Database (LHID) with disease histories of outpatients. Between 1997 and 2010, 980,157 people were insured and were then used as the database pool. The diagnoses and procedures recorded in the NHIRD are coded according to the International Classification of Diseases, Ninth Revision, Clinical Modification (ICD-9-CM). This study was approved by the Institutional Review Board of Tri-Service General Hospital and National Defense Medical Center in Taiwan (B-104-21).

### Sampled patients

**Figure 1:**
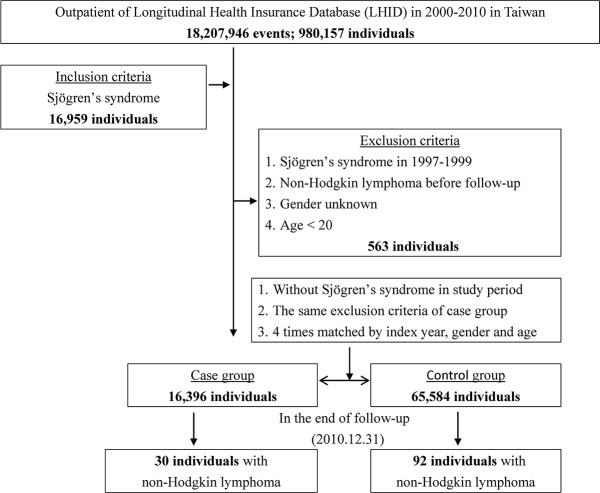
The flowchart of study sample selection from Taiwan's National Health Insurance Research Database

We conducted a population-based retrospective cohort study to determine the relationship between SS and the risk of developing NHL. Patients newly diagnosed with primary SS (ICD-9-CM code 710.2) between 2000 and 2010 were identified in the LHID (Figure 1). The SS diagnosis was based on ICD-9-CM codes, which were judged and determined by related specialists and physicians according to the standard clinical/laboratory/pathology criteria of the American-European Consensus Group [32]. The index date represents the date on which a patient was first diagnosed with SS. We excluded any patients with a history of NHL (ICD-9-CM code 200.0-200.7, 202.0-202.2, 202.7) prior to follow-up, had incomplete information with regard to gender, or were younger than 20 years old. For the non-SS cohort, we randomly chose patients without any history of SS from the LHID and frequency-matched them with the SS patients using a 4:1 ratio based on gender, age within an interval of five years, and the year of the index date. The exclusion criteria for the non-SS cohort were the same as the SS cohort.

### Outcome, comorbidities, and medications

The patients were followed-up from the index date until a diagnosis of NHL, withdrawal from the NHI program, or December 31, 2010. Preexisting comorbidities, such as diabetes (ICD-9-CM 250), hypertension (ICD-9-CM 401- 405), depression (ICD-9-CM 296.2, 296.3, 296.82, 300.4, and 311), stroke (ICD-9-CM 430-438), dementia (ICD-9-CM 290, 294.1, 331.0), and chronic kidney disease (ICD-9-CM 585), were also analyzed. Furthermore, we performed analysis on the subgroup that had salivary gland slices (NHI code 92021B) or were not in the SS group. Patients in the SS group that received immunomodulator agents (including hydroxychloroquine (NHI code AC42899100, AB50142100, AC48605100, AC49303100, AC55894100, AC55925100, AC58809100, BC22376100), azathioprine (NHI code BC09147100, AC45781100, AC49283100, BC22229100, BC25754100), cyclosporine (NHI code BC13027148, BC21188100, BC21189100, AC50140100, BC13049209, C242064CR), methotrexate (NHI code BC22726100, A025520100, BC16194100, BC18876221, BC18876229, BC18876248, BC18878221, BC21696212, BC21713229, BC22132229), and rituximab (NHI code KC00928248, KC00928229)) or not before being diagnosed with NHL were also analyzed.

### Statistical analysis

Using the Chi-square/Fisher exact test, we compared the distributions of categorical characteristics and baseline comorbidities between the SS group and non-SS control group. Differences in continuous variables between the cohorts were tested using the Student t-test. The cumulative incidence of NHL and the subtypes between the SS and non-SS cohorts was assessed using the Kaplan-Meier method, and the log-rank test was applied to compare the two cohorts. The incidence densities (per 10^5^ person-years) of NHL were calculated in both cohorts, and the hazard ratios (HR) categorized by gender, age, and such comorbidities as diabetes, hypertension, depression, stroke, dementia, and chronic kidney disease were calculated using Cox proportional-hazards regression models. All analyses were conducted using SAS Version 9.3 (SAS Institute Inc., Cary, NC), with the significance level set at 0.05 in two-tailed tests.

## RESULTS

### Patient demographics

**Figure 2:**
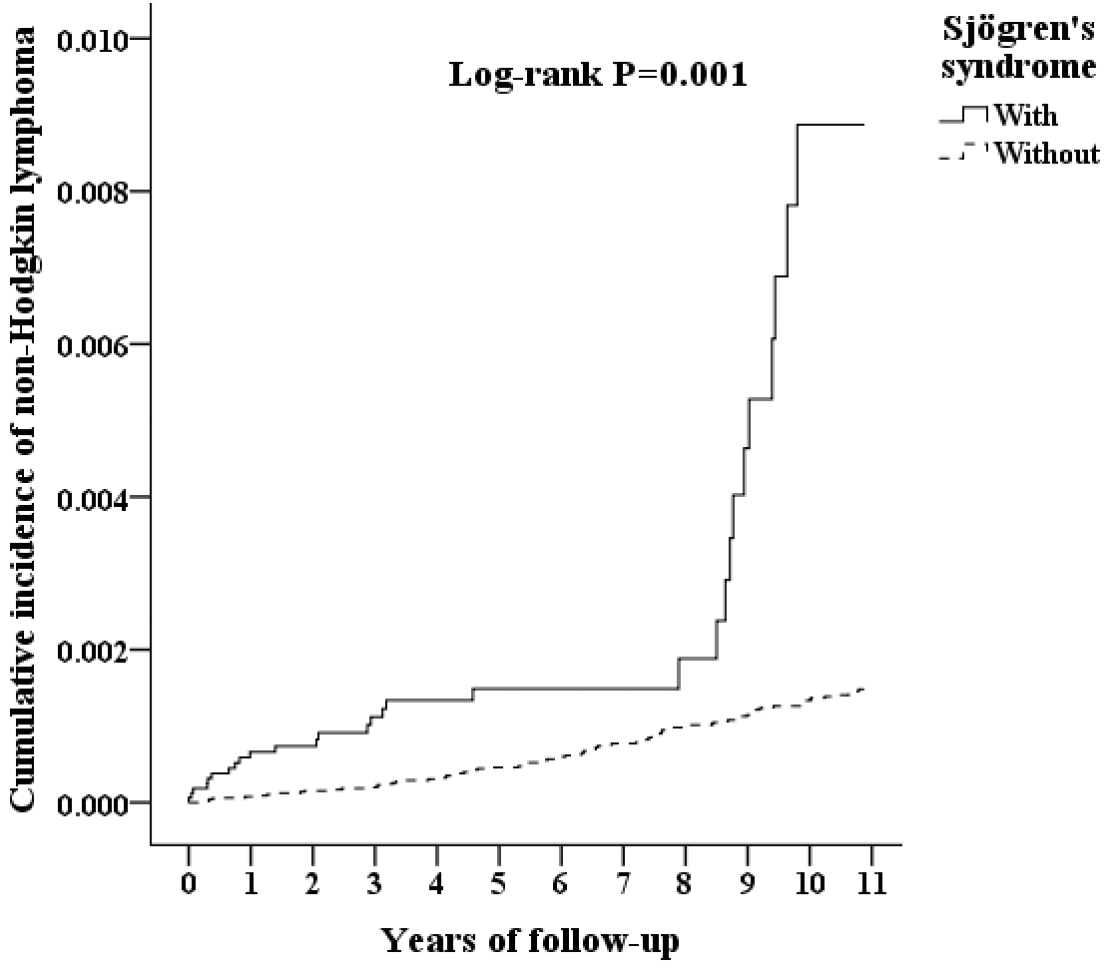
Kaplan-Meier for cumulative incidence of non-Hodgkin's lymphoma among patients aged 20 and over stratified by Sjögren's syndrome using the log-rank test

The SS group consisted of 16,396 individuals, and the non-SS group consisted of 65,584 individuals. The age distributions of the cohorts were 50.19±16.44 and 49.82±16.62 (Table 2), respectively. Women constituted 73.78% of the patients with SS. Diabetes, hypertension, and stroke were more prevalent in the non-SS group. The comorbidities, including diabetes, hypertension, depression, stroke, dementia, and chronic kidney disease, as well as age and gender, are all adjusted in Cox regression (Table 3). The Kaplan-Meier for cumulative incidence of NHL was 0.04% higher in the SS cohort than in the non-SS cohort with *P* = 0.001 (Figure 2). The mean durations until the development of NHL in the SS and non-SS cohorts were 4.54 (SD = 3.99, 0.01 to 10.97) and 6.27 (SD = 2.88, 0.27 to 10.96) years, respectively.

**Table 2:** Characteristics of patients with and without Sjögren's syndrome

Sjögren's syndrome	With (Case)		Without (Control)		*P*-value
Variables	*n*	%	*n*	%	
Total	16,396		65,584		
Female	12,097	73.78	48,388	73.78	0.999
Age (years) (mean±SD)	50.19±16.44		49.82±16.62		0.935
Comorbidity					
Diabetes	218	1.70	1,943	2.96	<0.001
Hypertension	536	3.27	4,674	7.13	<0.001
Depression	68	0.41	210	0.32	0.071
Stroke	66	0.40	691	1.05	<0.001
Dementia	13	0.08	72	0.11	0.342
Chronic kidney disease	36	0.22	113	0.17	0.218

**Table 3:** Multivariable Analysis for non-Hodgkin's lymphoma at the end of follow-up by using Cox regression

Variables	Crude HR	95% CI	*P*-value	Adjusted HR	95% CI	*P*-value
Sjögren's syndrome	4.268	2.749- 6.625	<0.001	4.314	2.784 -6.685	<0.001
Gender (Male)	1.090	0.731 -1.626	0.671	1.041	0.697 -1.556	0.843
Age (years)	1.016	1.005 -1.027	0.005	1.022	1.010 -1.033	<0.001
Diabetes	1.008	0.470 -2.162	0.984	1.257	0.570 -2.771	0.571
Hypertension	0.478	0.223 -1.026	0.058	0.411	0.186 -1.009	0.051
Stroke	0.327	0.046 -2.338	0.265	0.315	0.044 -2.276	0.252

Abbreviations: HR= hazard ratio, CI = confidence interval, Adjusted HR: Adjusted variables including Sjögren's syndrome, gender, age, diabetes, hypertension, depression, stroke, dementia, and chronic kidney disease.

### Risk estimation

In this study, age and SS are the two independent risk factors of NHL. The risk of NHL increases with an adjusted HR of 1.022 (95% CI 1.010 - 1.033) by year of age. Among the SS cohort and the non-SS cohort, 30 and 92 patients, respectively, developed NHL, with the incidence rates being 43.42 per 100,000 Person-years and 13.54 per 100,000 Person-years, resulting in an adjusted HR of 4.314 (95% CI 2.784 - 6.685). The incidence rates of B-cell NHL (ICD-9-CM 200.0 - 200.5, 200.7, 202.0, 202.2) in the SS and non-SS groups were 40.53 and 11.62 per 100,000 Person-years, respectively, with an adjusted HR of 9.87 (95% CI 3.764 - 18.757). The incidence rates of T-cell NHL (ICD-9-CM 200.6, 202.1, 202.2, 202.7) in the SS and non-SS groups were 2.89 and 1.91 per 100,000 Person-years, respectively, with an adjusted HR of 3.675 (95% CI 1.045 - 5.724). The highest risk was diffused large B-cell lymphoma, with the incidence rates in the SS and non-SS groups being 13.03 and 0.29 per 100,000 Person-years, respectively, with an adjusted HR of 45.670 (95% CI 2.034 - 110.018) (Table 4).

**Table 4:** Factors of non-Hodgkin's lymphoma at the end of the follow-up period stratified by Cox regression

	Sjögren's syndrome			Non- Sjögren's syndrome			Ratio	Adjusted HR (95% CI)	P-value
Non-Hodgkin's lymphoma	Event	PYs	Rate	Event	PYs	Rate			
**Total**	**30**	**69,086.94**	**43.42**	**92**	**679,671.79**	**13.54**	**3.208**	**4.314 (2.784 – 6.865)**	**<0.001**
**B-cell type**	**28**	**69,086.94**	**40.53**	**79**	**679,671.79**	**11.62**	**3.487**	**9.870 (3.764 – 18.757)**	**0.003**
Diffused large B-cell lymphoma	9	69,086.94	13.03	2	679,671.79	0.29	44.271	45.670 (2.034 – 110.018)	0.010
**T-cell type**	**2**	**69,086.94**	**2.89**	**13**	**679,671.79**	**1.91**	**1.514**	**3.675 (1.045 – 5.724)**	**0.014**

Abbreviations: PYs = Person-years; Ratio = Rate in cases ÷ Rate in controls; Adjusted HR = Adjusted Hazard ratio: Adjusted for all the variables of gender, age, and comorbidities, including diabetes, hypertension, depression, stroke, dementia, and chronic kidney disease; CI = confidence interval.

### Sensitivity analysis

In the SS group of 16,396 individuals, 66 individuals had salivary gland slices without NHL development; meanwhile, of the 16,330 individuals that did not have salivary gland slices, 30 individuals developed NHL. Of the SS group, 128 individuals received immunomodulator agent therapy without NHL development. All 30 individuals that developed NHL were in the group of SS patients that had not received immunomodulator agents.

Finally, we conducted sensitivity analyses to assess the relationships between SS and the risk of developing NHL at different follow-up durations (Table 5). Our findings indicate that, compared to the control cohort, the SS cohort was correlated with a significantly higher risk of developing NHL as the follow-up duration increased. The adjusted HRs were 3.456 (95% CI = 2.189 - 4.765) for follow-up periods less than one year, 4.325 (95% CI = 2.886 - 6.097) for follow-up periods between one and two years, and 5.979 (95% CI = 2.912 - 8.774) for follow-up periods of more than two years.

**Table 5:** Durations from diagnosis of Sjögren's syndrome to development of non-Hodgkin's lymphoma

	Sjögren's syndrome			Non- Sjögren's syndrome			Adjusted HR	95%CI	*P*-value
Follow-up durations	Event	PYs	Rate (per 10^5^ PYs)	Event	PYs	Rate (per 10^5^ PYs)			
**Total**	30	69,086.94	43.42	92	679,671.79	13.54	4.314	2.784 - 6.685	<0.001
<1	2	150.64	1,327.67	2	399.78	500.28	3.456	2.189 - 4.765	0.015
≧1, <2	4	224.79	1,779.44	4	610.99	654.68	4.325	2.886 - 6.097	0.003
≧2	24	68,711.51	34.93	86	678,661.02	12.67	5.979	2.912 - 8.774	<0.001

Abbreviations: PYs = Person-years; Ratio = Rate in cases ÷ Rate in controls; Adjusted HR = Adjusted Hazard ratio: Adjusted for all the variables of gender, age, and comorbidities, including diabetes, hypertension, depression, stroke, dementia, and chronic kidney disease; CI = confidence interval.

## DISCUSSION

In this study, we investigated the occurrence of NHL among SS patients in Taiwan in a nationwide population database with complete follow-up assessment. Age and SS are independent risk factors of NHL. We found NHL risk to increase 2.2% per year of age. We found SS to be correlated with NHL, with a 4.3-fold increased risk of NHL than the normal population during the follow-up period. NHL subgroup analysis revealed a higher incidence of B-cell NHL than T-cell NHL, with the risk of both being significantly increased in the SS than non-SS groups. NHL subgroup analysis revealed that patients with diffused large B-cell lymphoma have a higher risk (HR = 45.67; 95% CI, 2.034 - 110.018) than the normal population. For the NHIRD collected through ICD-9-CM, the diagnostic term differed from the current World Health Organization (WHO) classification of hematopoietic and lymphoid tumors [38]. We summarized the classification into B-cell type (ICD-9-CM 200.0 - 200.5, 200.7, 202.0, 202.2) and T-cell type (ICD-9-CM 200.6, 202.1, 202.2, 202.7) and then found that diffused large B-cell lymphoma had a higher risk in the SS group compared to non-SS patients with adjusted HR 45.67. These findings agree with previous cohort studies, with diffuse large B-cell lymphoma, mucosa-associated lymphoid tissue lymphoma, and nodal marginal zone lymphoma being the most prevalent [5, 7, 9, 15, 21, 25]. These results may be associated with increased B-cell activity in SS patients.

The multi-step process between SS and lymphoma is still unclear. SS is considered to be strongly associated with B-cell dysregulation [39]. The B-cell is differentiated from hematopoietic stem cells in the bone marrow and then migrates through the blood to the secondary lymphoid organs for maturation. During the differentiation process, the immature B cells undergo positive selection and negative selection, in which B-cell receptors (BCR) have an important role [40]. Such transcription factors as Ikaros, PU.1, E2A, early B cell factor, and Pax5 all promote B cell commitment and differentiation [41]. In general, autoreactive B cells go through clonal deletion, receptor editing, anergy, or ignorance during negative selection [42]. The DNA binding transcription factor, such as the nuclear factor kappa binding molecule (NFκB), promotes the expression of different gene sets and induces immunogenic proliferation and antibody production, while the nuclear factor of activated T cells (NFATc) are instrumental to inhibiting genes [42]. Most mature B cells respond to T-cell dependent foreign antigens on follicular dendritic cells at lymph nodes and the spleen and then differentiate into plasma cells or enter the germinal center (GC) [43]. Meanwhile, CD-5 positive B cells, Marginal zone B cells, and GC B cells are differentiated into plasma cells through adaptive immunity and contribute to the pool of circulating natural antibodies [44].

The B-cell activating factor (BAFF), also called the B-lymphocyte stimulator (BLyS), is a part of the TNF-family and promotes the maturation of transitional B cells into the B cell [45]. BAFF and BAFF receptor (BAFF-R) signaling is also required for survival in marginal zones and follicular B cells [46]. One animal study using mice proved that the autoantigen binding B cells increased dependence on BAFF for survival [47]. In humans, BAFF-R is expressed on almost all B cells, except for bone marrow plasma cells and a small subset of resting T cells [48, 49]. BAFF-R interacts with BCR in the regulation of deletion, anergy, and the survival of B cells through PI3K signaling pathways, NFκB pathways, modulating the transcription factor Pax5, and inhibiting additively apoptotic pathways [48, 50–52]. These interactions can play an important role in lymphoma progression.

Seronegative SS patients have less hyperactive B cells, including hypergammaglobulinemia, and less positivity on antinuclear antibodies (ANA) and rheumatoid factor (RF) [53]. Regarding the subgroup analysis of SS patients, we found that candidates that underwent lip biopsies are mostly seronegative SS, thus fulfilling the 2002 AECG criteria. Whether the immunomodulator is related to the risk of lymphoma remains a controversial issue [54, 55]. In the analysis of the British Society for Rheumatology Biologics Register - Rheumatoid Arthritis between 2001-2009, an increasing risk of lymphoma was not found in patients with rheumatoid arthritis who received anti-tumor necrosis factor therapy [56].

Although the subset is small, no SS patients that had salivary gland slices or immunomodulator developed NHL during the follow-up period in our cohort study for sensitivity analysis. We can hypothesize that SS patients who require salivary gland slices or received immunomodulator agents are correlated with a lower risk of lymphoma than other SS patients. This topic requires further investigation in the future.

Our study enrolled a large sample size from the nationwide population-based cohort, which enhances the statistical power of this study. The nationwide database used has a very high coverage rate, so almost all the patients' follow-up data are available. The population-based data represent Taiwan's general population.

As a retrospective cohort study, this study has lower statistical quality. Bias from unknown confounders may have affected our results, and a well-designed randomized prospective control study is still necessary to help establish a causal relationship. The current investigation was limited since NHIRD only provided age, gender, time of event, diagnosis, and management. The NHIRD does not provide such information as patients' subjective or objective history, physical examinations, primary lab data, or imaging. Since the NHIRD was collected through ICD-9-CM, differences between the clinically used WHO classification and ICD-9-CM may have resulted in bias. We summarized the classification into B cell NHL (ICD-9-CM 200.0 - 200.5, 200.7, 202.0, 202.2) and T cell NHL (ICD-9-CM 200.6, 202.1, 202.2, 202.7). Lymphoma was found from clinical symptoms of mass lesion and the pathology from biopsy. A malignancy screen was not routinely performed on all patients. If the patient was not aware of the symptoms or the patient did not seek medical assistance from a registered NHI clinic, the diagnosis of malignancy, including lymphoma, would be underestimated. NHL seems to occur in a very small number of patients (30/16396 cases and 92/65,584 controls). While the HR of 4.314 was found to be statistically significant, the clinical relevance at such a low incidence needs to be carefully interpreted. We used salivary gland slices to represent that seronegative patients may have a selection bias. The population of SS patients may also have been underestimated since the initial symptoms may not have been serious enough for the patient to consult a doctor. AECG updated its criteria in 2012, at which point it reduced items [57]. Two out of three criteria were required to be considered serology positive (anti-Ro, anti-La, or a combination of rheumatoid factor and ANA ≥1:320); Ocular staining score ≥ 3; and labial salivary gland biopsies with lymphocytic focus score ≥ 1 focus/4mm2 [57]. These updated criteria better highlight the importance of biopsy and serology.

## CONCLUSIONS

Sjögren syndrome increases the risk of NHL, particularly in B-cell NHL especially diffused large B-cell lymphoma. The anti-Ro/La negative Sjögren syndrome patients that had salivary gland slices were found to have less risk of developing lymphoma. The issue of prescribing immunomodulator agent to patients with SS who do not have extraoral and extraglandular manifestations is controversial. The clinical practice guidelines from Sjögren's Syndrome Foundation suggested that hydroxychloroquine is the first line therapy for patients with musculoskeletal pain [58]. Aggressively managing Sjögren syndrome disease activity may potentially decrease the risk of lymphoma over time. We recommend that patients at a higher risk upon diagnosis of SS have close follow-up and aggressive treatment.

## SUPPLEMENTARY MATERIALS TABLE



## References

[1] Singh AG, Singh S, Matteson EL (2016). Rate, risk factors and causes of mortality in patients with Sjogren's syndrome: a systematic review and meta-analysis of cohort studies. Rheumatology (Oxford).

[2] Theander E, Manthorpe R, Jacobsson LT (2004). Mortality and causes of death in primary Sjogren's syndrome: a prospective cohort study. Arthritis Rheum.

[3] Retamozo S, Gheitasi H, Quartuccio L, Kostov B, Corazza L, Bové A, Sisó-Almirall A, Gandía M, Ramos-Casals M, De Vita S (2016). Cryoglobulinaemic vasculitis at diagnosis predicts mortality in primary Sjögren syndrome: analysis of 515 patients. Rheumatology (Oxford).

[4] Baldini C, Pepe P, Quartuccio L, Priori R, Bartoloni E, Alunno A, Gattamelata A, Maset M, Modesti M, Tavoni A, De Vita S, Gerli R, Valesini G (2014). Primary Sjogren's syndrome as a multi-organ disease: impact of the serological profile on the clinical presentation of the disease in a large cohort of Italian patients. Rheumatology (Oxford).

[5] Voulgarelis M, Ziakas PD, Papageorgiou A, Baimpa E, Tzioufas AG, Moutsopoulos HM (2012). Prognosis and outcome of non-Hodgkin lymphoma in primary Sjogren syndrome. Medicine (Baltimore).

[6] Theander E, Vasaitis L, Baecklund E, Nordmark G, Warfvinge G, Liedholm R, Brokstad K, Jonsson R, Jonsson MV (2011). Lymphoid organisation in labial salivary gland biopsies is a possible predictor for the development of malignant lymphoma in primary Sjogren's syndrome. Ann Rheum Dis.

[7] Solans-Laque R, Lopez-Hernandez A, Bosch-Gil JA, Palacios A, Campillo M, Vilardell-Tarres M (2011). Risk, predictors, and clinical characteristics of lymphoma development in primary Sjogren's syndrome. Semin Arthritis Rheum.

[8] Martel C, Gondran G, Launay D, Lalloue F, Palat S, Lambert M, Ly K, Loustaud-Ratti V, Bezanahary H, Hachulla E, Jauberteau MO, Vidal E, Hatron PY (2011). Active immunological profile is associated with systemic Sjogren's syndrome. J Clin Immunol.

[9] Ekstrom Smedby K, Vajdic CM, Falster M, Engels EA, Martinez-Maza O, Turner J, Hjalgrim H, Vineis P, Seniori Costantini A, Bracci PM, Holly EA, Willett E, Spinelli JJ (2008). Autoimmune disorders and risk of non-Hodgkin lymphoma subtypes: a pooled analysis within the InterLymph Consortium. Blood.

[10] Valesini G, Priori R, Bavoillot D, Osborn J, Danieli MG, Del Papa N, Gerli R, Pietrogrande M, Sabbadini MG, Silvestris F, Valsecchi L (1997). Differential risk of non-Hodgkin's lymphoma in Italian patients with primary Sjogren's syndrome. J Rheumatol.

[11] Andonopoulos AP, Tiniakou M, Melachrinou M, Sfountouris H, Bounas A, Zervas C, Zoumbos NC (1997). Sjogren's syndrome in patients with newly diagnosed untreated non-Hodgkin's lymphoma. Rev Rhum Engl Ed.

[12] Talal N, Bunim JJ (1964). THE DEVELOPMENT OF MALIGNANT LYMPHOMA IN THE COURSE OF SJOEGREN's SYNDROME. Am J Med.

[13] Zintzaras E, Voulgarelis M, Moutsopoulos HM (2005). The risk of lymphoma development in autoimmune diseases: a meta-analysis. Arch Intern Med.

[14] Tzioufas AG, Boumba DS, Skopouli FN, Moutsopoulos HM (1996). Mixed monoclonal cryoglobulinemia and monoclonal rheumatoid factor cross-reactive idiotypes as predictive factors for the development of lymphoma in primary Sjogren's syndrome. Arthritis Rheum.

[15] Smedby KE, Hjalgrim H, Askling J, Chang ET, Gregersen H, Porwit-MacDonald A, Sundstrom C, Akerman M, Melbye M, Glimelius B, Adami HO (2006). Autoimmune and chronic inflammatory disorders and risk of non-Hodgkin lymphoma by subtype. J Natl Cancer Inst.

[16] Skopouli FN, Dafni U, Ioannidis JP, Moutsopoulos HM (2000). Clinical evolution, and morbidity and mortality of primary Sjogren's syndrome. Semin Arthritis Rheum.

[17] Pavlidis NA, Drosos AA, Papadimitriou C, Talal N, Moutsopoulos HM (1992). Lymphoma in Sjogren's syndrome. Med Pediatr Oncol.

[18] Lazarus MN, Robinson D, Mak V, Moller H, Isenberg DA (2006). Incidence of cancer in a cohort of patients with primary Sjogren's syndrome. Rheumatology (Oxford).

[19] Kauppi M, Pukkala E, Isomaki H (1997). Elevated incidence of hematologic malignancies in patients with Sjogren's syndrome compared with patients with rheumatoid arthritis (Finland). Cancer Causes Control.

[20] Kassan SS, Thomas TL, Moutsopoulos HM, Hoover R, Kimberly RP, Budman DR, Costa J, Decker JL, Chused TM (1978). Increased risk of lymphoma in sicca syndrome. Ann Intern Med.

[21] Baimpa E, Dahabreh IJ, Voulgarelis M, Moutsopoulos HM (2009). Hematologic manifestations and predictors of lymphoma development in primary Sjogren syndrome: clinical and pathophysiologic aspects. Medicine (Baltimore).

[22] Weng MY, Huang YT, Liu MF, Lu TH (2012). Incidence of cancer in a nationwide population cohort of 7852 patients with primary Sjogren's syndrome in Taiwan. Ann Rheum Dis.

[23] Pertovaara M, Pukkala E, Laippala P, Miettinen A, Pasternack A (2001). A longitudinal cohort study of Finnish patients with primary Sjogren's syndrome: clinical, immunological, and epidemiological aspects. Ann Rheum Dis.

[24] Davidson BK, Kelly CA, Griffiths ID (1999). Primary Sjogren's syndrome in the North East of England: a long-term follow-up study. Rheumatology (Oxford).

[25] Sutcliffe N, Inanc M, Speight P, Isenberg D (1998). Predictors of lymphoma development in primary Sjogren's syndrome. Semin Arthritis Rheum.

[26] Brito-Zeron P, Ramos-Casals M, Bove A, Sentis J, Font J (2007). Predicting adverse outcomes in primary Sjogren's syndrome: identification of prognostic factors. Rheumatology (Oxford).

[27] Ramos-Casals M, Brito-Zeron P, Yague J, Akasbi M, Bautista R, Ruano M, Claver G, Gil V, Font J (2005). Hypocomplementaemia as an immunological marker of morbidity and mortality in patients with primary Sjogren's syndrome. Rheumatology (Oxford).

[28] Ioannidis JP, Vassiliou VA, Moutsopoulos HM (2002). Long-term risk of mortality and lymphoproliferative disease and predictive classification of primary Sjogren's syndrome. Arthritis Rheum.

[29] Voulgarelis M, Dafni UG, Isenberg DA, Moutsopoulos HM (1999). Malignant lymphoma in primary Sjogren's syndrome: a multicenter, retrospective, clinical study by the European Concerted Action on Sjogren's Syndrome. Arthritis Rheum.

[30] Anaya JM, McGuff HS, Banks PM, Talal N (1996). Clinicopathological factors relating malignant lymphoma with Sjogren's syndrome. Semin Arthritis Rheum.

[31] Quartuccio L, Isola M, Baldini C, Priori R, Bartoloni Bocci E, Carubbi F, Maset M, Gregoraci G, Della Mea V, Salvin S, De Marchi G, Luciano N, Colafrancesco S (2014). Biomarkers of lymphoma in Sjogren's syndrome and evaluation of the lymphoma risk in prelymphomatous conditions: results of a multicenter study. J Autoimmun.

[32] Vitali C, Bombardieri S, Jonsson R, Moutsopoulos HM, Alexander EL, Carsons SE, Daniels TE, Fox PC, Fox RI, Kassan SS, Pillemer SR, Talal N, Weisman MH (2002). Classification criteria for Sjogren's syndrome: a revised version of the European criteria proposed by the American-European Consensus Group. Ann Rheum Dis.

[33] Groom J, Kalled SL, Cutler AH, Olson C, Woodcock SA, Schneider P, Tschopp J, Cachero TG, Batten M, Wheway J, Mauri D, Cavill D, Gordon TP (2002). Association of BAFF/BLyS overexpression and altered B cell differentiation with Sjogren's syndrome. J Clin Invest.

[34] Cheema GS, Roschke V, Hilbert DM, Stohl W (2001). Elevated serum B lymphocyte stimulator levels in patients with systemic immune-based rheumatic diseases. Arthritis Rheum.

[35] National Health Insurance Administration MoHaW, Taiwan, R.O.C. (2015). National Health Insurance in Taiwan 2015-2016 Annual Report.

[36] Liu FC, Huang WY, Lin TY, Shen CH, Chou YC, Lin CL, Lin KT, Kao CH (2015). Inverse Association of Parkinson Disease With Systemic Lupus Erythematosus: A Nationwide Population-based Study. Medicine (Baltimore).

[37] Hsing AW, Ioannidis JP (2015). Nationwide Population Science: Lessons From the Taiwan National Health Insurance Research Database. JAMA Intern Med.

[38] Swerdlow SH, Campo E, Pileri SA, Harris NL, Stein H, Siebert R, Advani R, Ghielmini M, Salles GA, Zelenetz AD, Jaffe ES (2016). The 2016 revision of the World Health Organization classification of lymphoid neoplasms. Blood.

[39] Routsias JG, Goules JD, Charalampakis G, Tzima S, Papageorgiou A, Voulgarelis M (2013). Malignant lymphoma in primary Sjogren's syndrome: an update on the pathogenesis and treatment. Semin Arthritis Rheum.

[40] Lam KP, Kuhn R, Rajewsky K (1997). *In vivo* ablation of surface immunoglobulin on mature B cells by inducible gene targeting results in rapid cell death. Cell.

[41] Nutt SL, Kee BL (2007). The transcriptional regulation of B cell lineage commitment. Immunity.

[42] Goodnow CC (2001). Pathways for self-tolerance and the treatment of autoimmune diseases. Lancet.

[43] LeBien TW, Tedder TF (2008). B lymphocytes: how they develop and function. Blood.

[44] Radbruch A, Muehlinghaus G, Luger EO, Inamine A, Smith KG, Dorner T, Hiepe F (2006). Competence and competition: the challenge of becoming a long-lived plasma cell. Nat Rev Immunol.

[45] Tussiwand R, Bosco N, Ceredig R, Rolink AG (2009). Tolerance checkpoints in B-cell development: Johnny B good. Eur J Immunol.

[46] Rauch M, Tussiwand R, Bosco N, Rolink AG (2009). Crucial role for BAFF-BAFF-R signaling in the survival and maintenance of mature B cells. PLoS One.

[47] Lesley R, Xu Y, Kalled SL, Hess DM, Schwab SR, Shu HB, Cyster JG (2004). Reduced Competitiveness of Autoantigen-Engaged B Cells due to Increased Dependence on BAFF. Immunity.

[48] Mackay F, Schneider P (2009). Cracking the BAFF code. Nat Rev Immunol.

[49] Tangye SG, Bryant VL, Cuss AK, Good KL (2006). BAFF, APRIL and human B cell disorders. Semin Immunol.

[50] Mackay F, Figgett WA, Saulep D, Lepage M, Hibbs ML (2010). B-cell stage and context-dependent requirements for survival signals from BAFF and the B-cell receptor. Immunol Rev.

[51] Stadanlick JE, Kaileh M, Karnell FG, Scholz JL, Miller JP, Quinn WJ, Brezski RJ, Treml LS, Jordan KA, Monroe JG, Sen R, Cancro MP (2008). Tonic B cell antigen receptor signals supply an NF-kappaB substrate for prosurvival BLyS signaling. Nat Immunol.

[52] Henley T, Kovesdi D, Turner M (2008). B-cell responses to B-cell activation factor of the TNF family (BAFF) are impaired in the absence of PI3K delta. Eur J Immunol.

[53] Quartuccio L, Baldini C, Bartoloni E, Priori R, Carubbi F, Corazza L, Alunno A, Colafrancesco S, Luciano N, Giacomelli R, Gerli R, Valesini G, Bombardieri S (2015). Anti-SSA/SSB-negative Sjogren's syndrome shows a lower prevalence of lymphoproliferative manifestations, and a lower risk of lymphoma evolution. Autoimmun Rev.

[54] Hasserjian RP, Chen S, Perkins SL, de Leval L, Kinney MC, Barry TS, Said J, Lim MS, Finn WG, Medeiros LJ, Harris NL, O'Malley DP (2009). Immunomodulator agent-related lymphoproliferative disorders. Mod Pathol.

[55] Shelton E, Laharie D, Scott FI, Mamtani R, Lewis JD, Colombel JF, Ananthakrishnan AN (2016). Cancer Recurrence Following Immune-suppressive Therapies in Patients With Immune-mediated Diseases: a Systematic Review and Meta-analysis. Gastroenterology.

[56] Mercer LK, Galloway JB, Lunt M, Davies R, Low ALS, Dixon WG, Watson KD, Symmons DPM, Hyrich KL, BSRBR Control Centre Consortium (2012). The Risk of Lymphoma in Patients Receiving Anti-Tumor Necrosis Factor Therapy for Rheumatoid Arthritis: Results From the British Society for Rheumatology Biologics Register - Rheumatoid Arthritis. The American College of Rheumatology and the Association of Rheumatology Health Professionals 2012 Annual Meeting.

[57] Shiboski S, Shiboski C, Criswell L, Baer A, Challacombe S, Lanfranchi H, Schiødt M, Umehara H, Vivino F, Zhao Y, Dong Y, Greenspan D, Heidenreich A (2012). American College of Rheumatology Classification Criteria for Sjögren's Syndrome: A Data-Driven, Expert Consensus Approach in the SICCA Cohort. Arthritis Care Res (Hoboken).

[58] Vivino FB, Carsons SE, Foulks G, Daniels TE, Parke A, Brennan MT, Forstot SL, Scofield RH, Hammitt KM (2016). New Treatment Guidelines for Sjogren's Disease. Rheum Dis Clin North Am.

