# Supplementary material for: Predictable biomarkers of developing lymphoma in patients with Sjögren syndrome: a nationwide population-based cohort study
Source: Oncotarget. 2017 Feb 4;8(30):50098–108. doi: 10.18632/oncotarget.15100 (PMC5564832; doi:10.18632/oncotarget.15100)
Supplement: Supplementary file 1 [file oncotarget-08-50098-s001.doc]

|  | **Sjögren's syndrome** | | | **Non- Sjögren's syndrome** | | | **Ratio** | **Adjusted HR (95% CI)** | **P** |
| --- | --- | --- | --- | --- | --- | --- | --- | --- | --- |
| **Non-Hodgkin’s lymphoma** | **Event** | **PYs** | **Rate** | **Event** | **PYs** | **Rate** |
| Diffused large B-cell lymphoma | 9 | 69,086.94 | 13.03 | 2 | 679,671.79 | 0.29 | 44.271 | 45.670 (2.034 – 110.018) | 0.010 |
| Lymphosarcoma | 4 | 69,086.94 | 5.79 | 13 | 679,671.79 | 1.91 | 3.027 | 9.756 (2.034 – 30.124) | 0.024 |
| Nodular lymphoma | 8 | 69,086.94 | 11.58 | 28 | 679,671.79 | 4.12 | 2.811 | 5.746 (1.298 – 11.754) | 0.007 |
| Reticulosarcoma | 6 | 69,086.94 | 8.68 | 23 | 679,671.79 | 3.38 | 2.566 | 5.614 (1.706 – 18.476) | 0.005 |
| Burkitt's tumor or lymphoma | 1 | 69,086.94 | 1.45 | 13 | 679,671.79 | 1.91 | 0.757 | 1.001 (0.310 – 3.226) | 0.958 |
| Mycosis fungoides | 1 | 69,086.94 | 1.45 | 13 | 679,671.79 | 1.91 | 0.757 | 1.001 (0.310 – 3.229) | 0.958 |
| Marginal zone lymphoma | 0 | 69,086.94 | 0.00 | 0 | 679,671.79 | 0.00 | - | - | - |
| Mantle cell lymphoma | 0 | 69,086.94 | 0.00 | 0 | 679,671.79 | 0.00 | - | - | - |
| Primary central nervous system lymphoma | 0 | 69,086.94 | 0.00 | 0 | 679,671.79 | 0.00 | - | - | - |
| Anaplastic large cell lymphoma | 0 | 69,086.94 | 0.00 | 0 | 679,671.79 | 0.00 | - | - | - |
| Sézary's disease | 0 | 69,086.94 | 0.00 | 0 | 679,671.79 | 0.00 | - | - | - |
| Peripheral T-cell lymphoma | 1 | 69,086.94 | 1.45 | 0 | 679,671.79 | 0.00 | - | - | - |

Supplementary table 1. Subtypes of non-Hodgkin’s lymphoma at the end of the follow-up period stratified by Cox regression.

PYs = Person-years; Ratio = Rate in cases ÷ Rate in controls; Adjusted HR = Adjusted Hazard ratio: Adjusted for all the variables of gender, age, and comorbidities, including diabetes, hypertension, depression, stroke, dementia, and chronic kidney disease; CI = confidence interval.
